# Imaging of thoracic tuberculosis: pulmonary and extrapulmonary

**DOI:** 10.1093/bjro/tzae031

**Published:** 2024-09-23

**Authors:** Nuttaya Pattamapaspong, Thanat Kanthawang, Wilfred C G Peh, Nadia Hammami, Mouna Chelli Bouaziz, Mohamed Fethi Ladeb

**Affiliations:** Department of Radiology, Faculty of Medicine, Chiang Mai University, Chiang Mai 50200, Thailand; Department of Radiology, Faculty of Medicine, Chiang Mai University, Chiang Mai 50200, Thailand; Department of Diagnostic Radiology, Khoo Teck Puat Hospital, Singapore 768828, Republic of Singapore; Department of Neuroradiology, National Institute of Neurology Mongi Ben Hamida, Baab Saadoun 1007, Tunis, Tunisia; Department of Radiology, MT Kassab Institute of Orthopaedics, Ksar Said 2010, Tunis, Tunisia; Faculty of Medicine of Tunis, Tunis-El Manar University, Tunis, Tunisia; Department of Radiology, MT Kassab Institute of Orthopaedics, Ksar Said 2010, Tunis, Tunisia; Faculty of Medicine of Tunis, Tunis-El Manar University, Tunis, Tunisia

**Keywords:** breast tuberculosis, cardiac tuberculosis, chest wall tuberculosis, extrapulmonary tuberculosis, pulmonary tuberculosis, thoracic tuberculosis

## Abstract

Tuberculosis (TB) remains the leading cause of death from a single infectious agent globally, despite being a potentially curable disease. This disease typically affects the lungs but may involve many extrapulmonary sites, especially in patients with risk factors such as HIV infection. The clinical features of extrapulmonary TB may mimic many different disease entities, particularly at less common thoracic sites such as the heart, chest wall, and breast. Imaging has an important role in the early diagnosis of TB, helping to detect disease, guide appropriate laboratory investigation, demonstrate complications, and monitor disease progress and response to treatment. Imaging supports the clinical objective of achieving effective treatment outcome and complication prevention. This review aims to highlight the imaging spectrum of TB affecting both pulmonary and extrapulmonary sites in the thorax. We also briefly provide key background information about TB, such as epidemiology, pathogenesis, and diagnosis.

## Introduction

Despite being curable, tuberculosis (TB) was the second leading cause of death from a single infectious agent, after coronavirus disease (COVID), in 2022. Worldwide, approximately 10.6 million individuals contracted TB disease, causing an estimated 1.3 million deaths in people negative for HIV, with an additional 167 000 deaths among those who were HIV-positive.^1^ About a quarter of the world’s population are infected with *Mycobacterium tuberculosis*, with 5%-10% going on to develop TB disease during their lifetime. Eighty-five percent of people who develop TB disease can be successfully treated with standard drug regimens, with shorter treatment regimens for those with TB infection.^1^ Unfortunately, the COVID-19 pandemic reversed years of progress made in providing essential TB services, with global milestones and targets being mostly off-track.^1^

TB typically infects the lungs, giving rise to pulmonary TB, but also affects many extrapulmonary sites. The clinical features of TB, particularly extrapulmonary TB, may be non-specific. Imaging has an important role in its early diagnosis, particularly in patients presenting with non-specific symptoms. It helps to detect disease, guide appropriate laboratory investigation, confirm the diagnosis, and monitor disease progress and response to treatment. Imaging supports the clinical aim of achieving effective treatment outcome and prevention of complications. Delayed or inappropriate diagnosis and treatment can result in disease spread and development of multidrug-resistant variants. In this review, we aim to highlight the imaging spectrum of TB affecting both pulmonary and extrapulmonary sites within the thorax. We also briefly provide key background information such as epidemiology, pathogenesis, clinical presentations, and diagnosis, in relation to the thorax.

## Epidemiology

In 2022, the vast majority of people who developed TB lived in the World Health Organization (WHO) regions of South-East Asia (46%), Africa (23%), and the Western Pacific (18%), with smaller proportions in the Eastern Mediterranean (8.1%), Americas (3.1%), and Europe (2.2%).^1^ Risk factors for contracting TB infection and progression to active TB disease can be broadly classified into host and environmental factors.^2^^,^^3^ Host factors include immunosuppressed states (eg, HIV infection, chronic steroid use, diabetes mellitus, chronic renal disease, malnutrition), substance abuse (eg, drug abuse, tobacco use, chronic alcoholism), and other systemic conditions (eg, malignancy, chronic obstructive pulmonary disease).^3^ Environmental risk factors include close contact with persons who are smear-positive for pulmonary TB, living in endemic countries, being in an immigrant community, and working in healthcare or laboratories. The probability of developing TB disease is much higher among people living with HIV, with Africa having the highest proportion of TB-HIV co-infection.^1^ People living with HIV are 14-18 times more likely to develop TB disease than people without HIV.^4^

## Pathogenesis

TB is an airborne disease caused by the *M tuberculosis* complex, comprising *M tuberculosis* and 7 closely related mycobacterial species, including *Mycobacterium africanum* and *Mycobacterium bovis*.^5^ The differences of infective mycobacterial species lead to variability in epidemiology, mode of transmission, affected organs and disease virulence. Globally, majority of TB cases are due to *M tuberculosis*. *Mycobacterium africanum* is mostly restricted to West Africa, causing up to 50% of TB cases.^6^^,^^7^*Mycobacterium bovis* may cause TB in domestic and wild animals, with resultant incidental human infections, particularly in developing countries. Humans are infected primarily through the consumption of animal products, such as unpasteurized milk or contaminated raw meat.^8^ In developing countries, the human bovine TB incidence is about 10%, contributing to 2.1% of pulmonary TB and 9.4% of extrapulmonary TB.^9^ In developed countries, the incidence of human infections due to *M bovis* has markedly decreased, because of successful implementation of bovine TB control programmes in animals.^8^ Besides different rates of disease virulence, the various mycobacterial strains are also associated with different rates of extrapulmonary disease.^10^ Both *M africanum* and *M bovis* carry an increased risk of extrapulmonary TB involvement as the primary site, more commonly than in *M tuberculosis*.^11^

*Mycobacterium tuberculosis* is primarily spread from persons with pulmonary TB disease, with transmission via inhalation of 1-5 micron-sized respiratory droplet nuclei containing *M tuberculosis* which reaches lung alveoli.^5^ The tuberculous bacilli are mostly phagocytosed and destroyed by host alveolar macrophages.^3^ Triggering of immune response leads to granuloma formation around the bacilli, keeping the bacilli contained and preventing spread of infection. This is known as latent TB infection (LTBI). Detection of LTBI is achieved by using interferon-gamma release assay (IGRA) or Mantoux tuberculin skin test (TST).^5^ However, a positive TST or IGRA result does not automatically imply LTBI, as individuals who eliminate the infection may still be TST or IGRA positive because of memory T-cell responses.^3^

In a minority of patients, the host’s immune system fails to eliminate the infection, allowing bacilli to multiply within alveolar macrophages. When the macrophages die, bacilli spread via lymphatics or bloodstream to the lung and other parts of the body, resulting in TB disease. How *M tuberculosis* accesses lung parenchyma is still not fully understood, with a number of postulated mechanisms.^10^ The precise mechanisms for extrapulmonary dissemination are also largely unknown, though certain host-pathogen interactions may be used by tuberculous bacilli to mediate latent induction and pathogen reactivation.^3^ Although extrapulmonary TB usually results from lymphohaematogenous spread of primary infection, it can sometimes occur without pulmonary involvement. Patients with LTBI usually progress to TB disease within the first 2 years of infection or have underlying risk factors. These patients, also known as TB cases, are typically symptomatic and are infectious to others.^5^

Extrapulmonary TB comprised 16% of 7.5 million incident cases notified globally in 2019, ranging from 8% in the WHO Western Pacific region to 24% in the Eastern Mediterranean region.^12^ Many environmental, host and microbial characteristics have been identified as risk factors for extrapulmonary TB.^13^ Patients with TB-HIV co-infections have higher incidences of extrapulmonary manifestations, latent disease reactivation and disseminated disease. Among TB cases in people living with HIV, 25% have extrapulmonary TB, with lymph nodes being the most frequent site.^14^ Miliary TB is a serious form of extrapulmonary TB where tuberculous bacilli disseminate via the bloodstream to all parts of the body, causing TB disease in multiple sites. In the preantibiotic era, miliary TB occurred most commonly in infants and young children below 5 years, particularly in TB-endemic countries. More recently, it has been increasingly recognized in severely immunocompromised adults, particularly with advanced HIV infection.^5^^,^^15^

## Diagnosis

Diagnosing TB infection and distinguishing LTBI from TB disease are key to instituting appropriate treatment. The 2 tests currently used for detecting *M tuberculosis* infection are IGRA or Mantoux TST, helping to differentiate people infected with *M tuberculosis* from those who are not infected. However, both of these tests are not able to differentiate LTBI from TB disease. A negative test also does not exclude the diagnosis of LTBI or TB disease.^5^

Chest radiographs are useful for suggesting diagnosis of pulmonary TB disease, particularly if the findings are typical. Chest radiographs are also helpful in excluding pulmonary TB disease in a person with a positive IGRA or TST but who is asymptomatic.^5^ The diagnosis and treatment of extrapulmonary TB are challenging, requiring a targeted imaging approach. CT is usually utilized to further evaluate pulmonary, thoracic, abdominal, urogenital, and head and neck TB. MRI is the modality of choice for assessing TB of the brain, spine, and musculoskeletal system.^18^F-fluorodeoxyglucose positron-emission tomography (^18^F-FDG PET)/CT has an increasing role in the detection of multifocal tuberculous lesions and assessment of the response to treatment.^16^ Ultrasonography and CT may be used to guide diagnostic and therapeutic aspirations and drainages, as well as biopsies for histopathological and bacteriological confirmation of TB.

The definitive diagnosis of TB is made by isolation of *M tuberculosis* in a body sample, either from sputum or tissue of affected sites.^3^^,^^5^ Optimal bacterial examination consists of acid-fast bacilli smear, nucleic acid amplification test (NAAT), and specimen culture and identification of *M tuberculosis*. This is followed by drug susceptibility testing using growth-based and molecular methods.^5^ Unlike pulmonary TB, sites affected by extrapulmonary TB are frequently biopsied as they are often pauci-bacillary with negative cultures being frequent. Typical histopathological features of tuberculous granulomas strongly support the diagnosis of TB.^17^

The WHO has recognized that the effective management of TB requires both rapid diagnosis of TB and rapid detection of drug resistance, particularly in resource-poor regions. Currently, WHO is in a transitory period where the previous product-specific recommendations for rapid tests (eg, Xpert MTB/RIF) are being changed to class-based recommendations (eg, low complexity automated NAATs). Xpert MTB/RIF assay (Cepheid, Sunnyvale, USA) is one of the WHO-approved rapid diagnostic tests that can accurately detect both TB and rifampicin resistance in less than 2 h. The Xpert MTB/RIF assay rapid test is easier to handle compared to conventional NAATs. It is cartridge-based and uses real-time polymerase chain reaction on the GeneXpert platform to identify *M tuberculosis* and mutations associated with RIF resistance directly from sputum specimens within an automated self-contained test unit.^18^ Of 6.2 million people worldwide diagnosed with pulmonary TB in 2022, only 63% were bacteriologically confirmed, with regional variations (ranging from 56% in the Eastern Mediterranean to 79% in the Americas). Of those without a confirmatory laboratory diagnosis of TB disease, a presumptive diagnosis known as “clinically diagnosed TB” can be made, with institution of treatment on this basis.^1^

## Pulmonary TB

### Pathophysiology and clinical features

Pulmonary TB is classically categorized into primary and postprimary TB. Both entities differ in genetic predisposition, host immune status, age of onset, clinical course, histopathology, and susceptibility to Bacillus Calmette–Guérin (BCG) vaccination.^19^ In primary TB, clinical disease onset occurs within 1 year of initial infection. On exposure to tuberculous bacilli, the early granulomatous response is called the Ghon focus, usually occurring in subpleural parenchyma. While the cellular immune response is on-going, bacilli can spread via lymphatics to hilar lymph nodes (forming a primary Ghon complex). In the majority of patients, the Ghon complex heals with fibrosis and calcifications within weeks to months, forming the Ranke complex.

About 5%-15% of people initially infected with TB progresses to active TB disease, which occurs when the immune system fails to contain *M tuberculosis* multiplication.^3^ The remainder develop LTBI but remain at risk for reinfection. In LTBI, the patients are asymptomatic, non-infectious, positive on TST or IGRA, and should undergo preventive TB therapy. Patients with active TB disease are symptomatic, infectious, usually have positive sputum smears, are culture-positive, and need multidrug therapy.^5^

The symptoms of pulmonary TB include persistent cough, haemoptysis, chest pain, evening fever, night sweats, fatigue, and weight loss.^5^ Physical examination is often negative, except when consolidation is extensive. Pleural effusion is often the only physical examination manifestation of primary TB. Although pulmonary TB can be viewed as a dynamic continuum from *M tuberculosis* infection to active disease, patients are usually categorized as having either LTBI or active TB disease for simplicity in clinical and public health settings. Individuals can advance or reverse positions, depending on changes in host immunity and comorbidities.^3^ Atypical clinical presentation in patients with impaired immunological response or comorbidities can cause diagnostic difficulty. Subclinical pulmonary TB may be unrecognized in children, particularly those with HIV co-infection.^20^ In elderly patients, chronic coughing from co-existing lung lesions such as chronic obstructive pulmonary disease or cancer can mask tuberculous co-infection.^21^

Postprimary TB refers to reactivation or reinfection with tuberculous bacilli. Reactivation (or endogenous reinfection) usually results from the breakdown of old quiescent pulmonary foci, usually more than a year after the initial infection. Reactivation may be due to a decrease in immune defences and is higher in patients with HIV infection and other risk factors. Reinfection may occur with a different strain, especially in endemic areas.^22^

### Imaging features

Imaging appearances of pulmonary TB depend on the patient’s immune status and often, imaging cannot differentiate primary from postprimary TB.^22^ At present, suggested terminology for active TB disease is “active TB”, rather than differentiation into classical primary and postprimary types.^23^^,^^24^ Although many features of primary and postprimary TB overlap, classic distinguishing features of postprimary TB include a predilection for the upper lobes, absence of lymphadenopathy, and cavitation. Radiologically, postprimary TB may manifest as parenchymal disease, airway involvement, and pleural extension.^25^ However, in immunosuppressed patients, postprimary TB may have a pattern of lower lung involvement, lymphadenopathy, and pleural effusions.^23^

**Figure 1.**
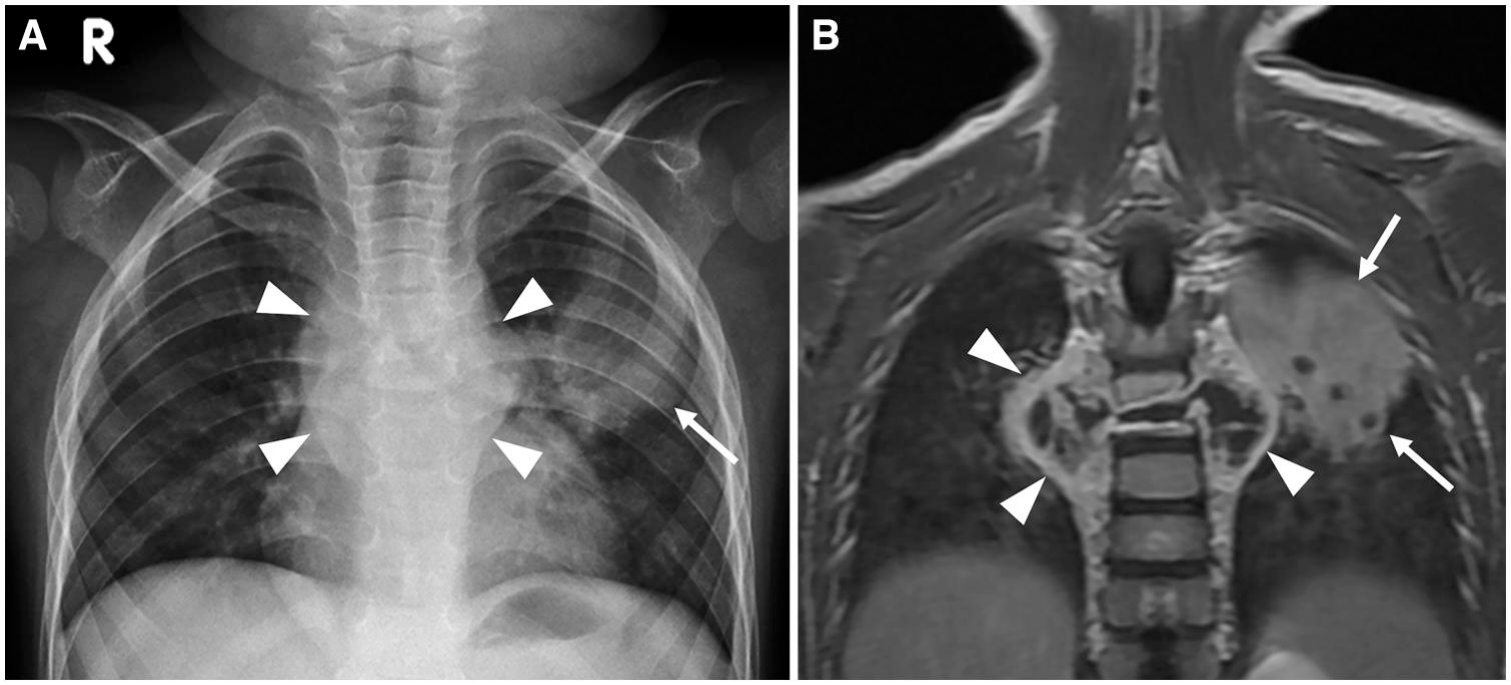
Primary pulmonary TB and thoracic spine TB in a 3-year-old girl. (A) Frontal chest radiograph shows patchy infiltration in the left upper lobe (white arrow) and paravertebral soft tissue swelling (white arrowheads). (B) Coronal contrast-enhanced T1-W MRI shows consolidation of the left upper lobe (white arrows) and destruction of the thoracic vertebrae with paravertebral abscesses (white arrowheads).

**Figure 2.**
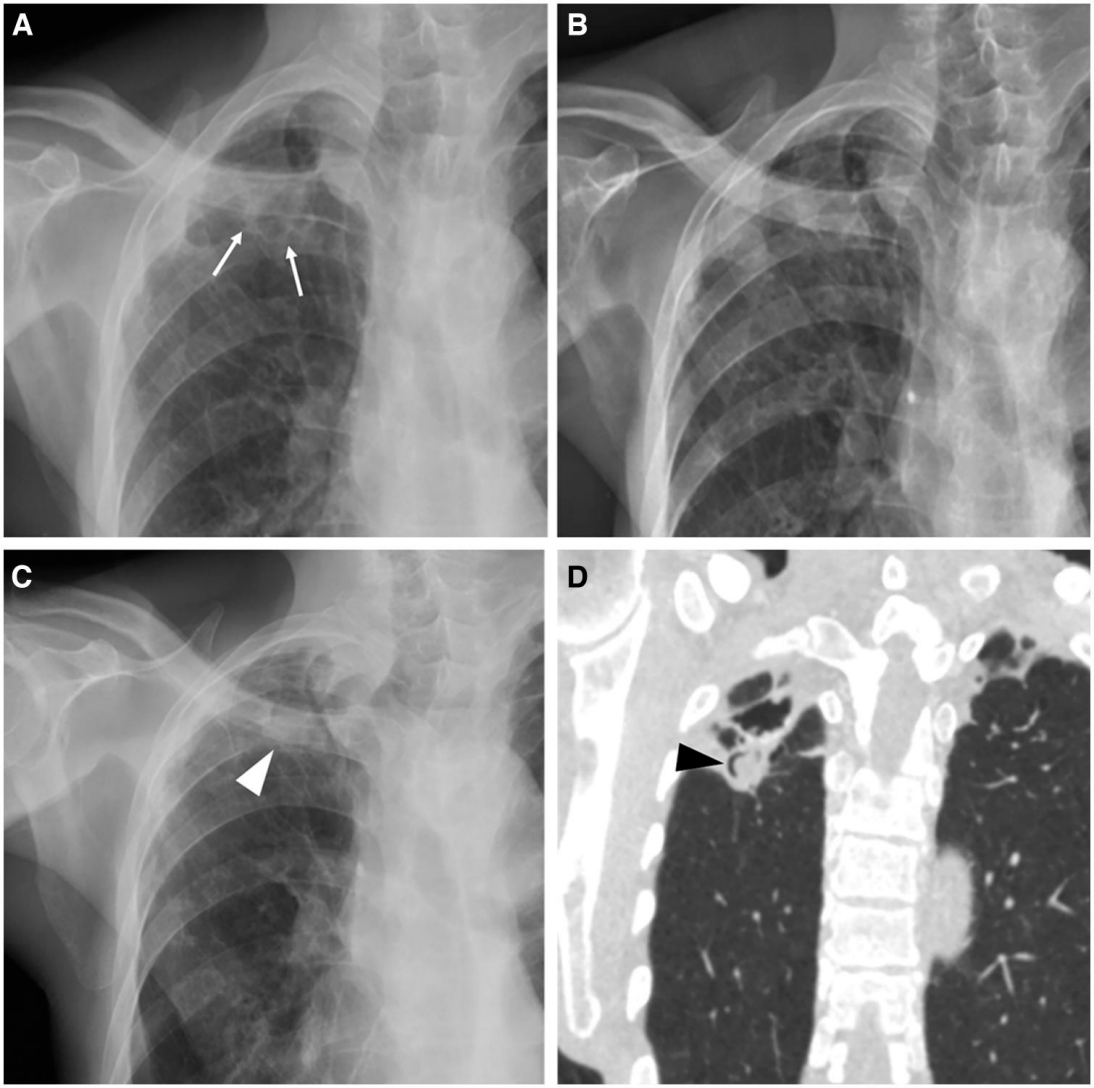
Postprimary pulmonary TB with cavitation and subsequent aspergilloma in an 88-year-old man. (A) Frontal chest radiograph shows a thick-walled cavitary lesion in the right upper lobe (white arrows). (B) Follow-up radiograph 2 years later shows healing with fibrosis. (C) Follow-up radiograph 5 years later shows development of a rounded opacity (white arrowhead). (D) Coronal CT image shows a fungal ball in the parenchymal cavity with an air crescent sign (black arrowhead). There is adjacent upper lobe fibrosis.

One of the earliest imaging features of active TB is consolidation, typically seen as dense, homogeneous parenchymal opacification in any lobe. Lower- and middle-lobe predominance is seen in adults. In children younger than 2 years, lobar or segmental atelectasis is frequent, usually involving anterior segment of the upper lobe or medial segment of the middle lobe.^25^ (Figure 1). With progressive disease, cavities form in up to 50% of patients (Figure 2A). These cavities are usually multiple and typically have thick irregular walls, becoming smooth and thin-walled with successful treatment.^26^ Resolution of parenchymal consolidation may take as long as 2 years, with resultant parenchymal scarring^22^ (Figure 2B).

**Figure 3.**
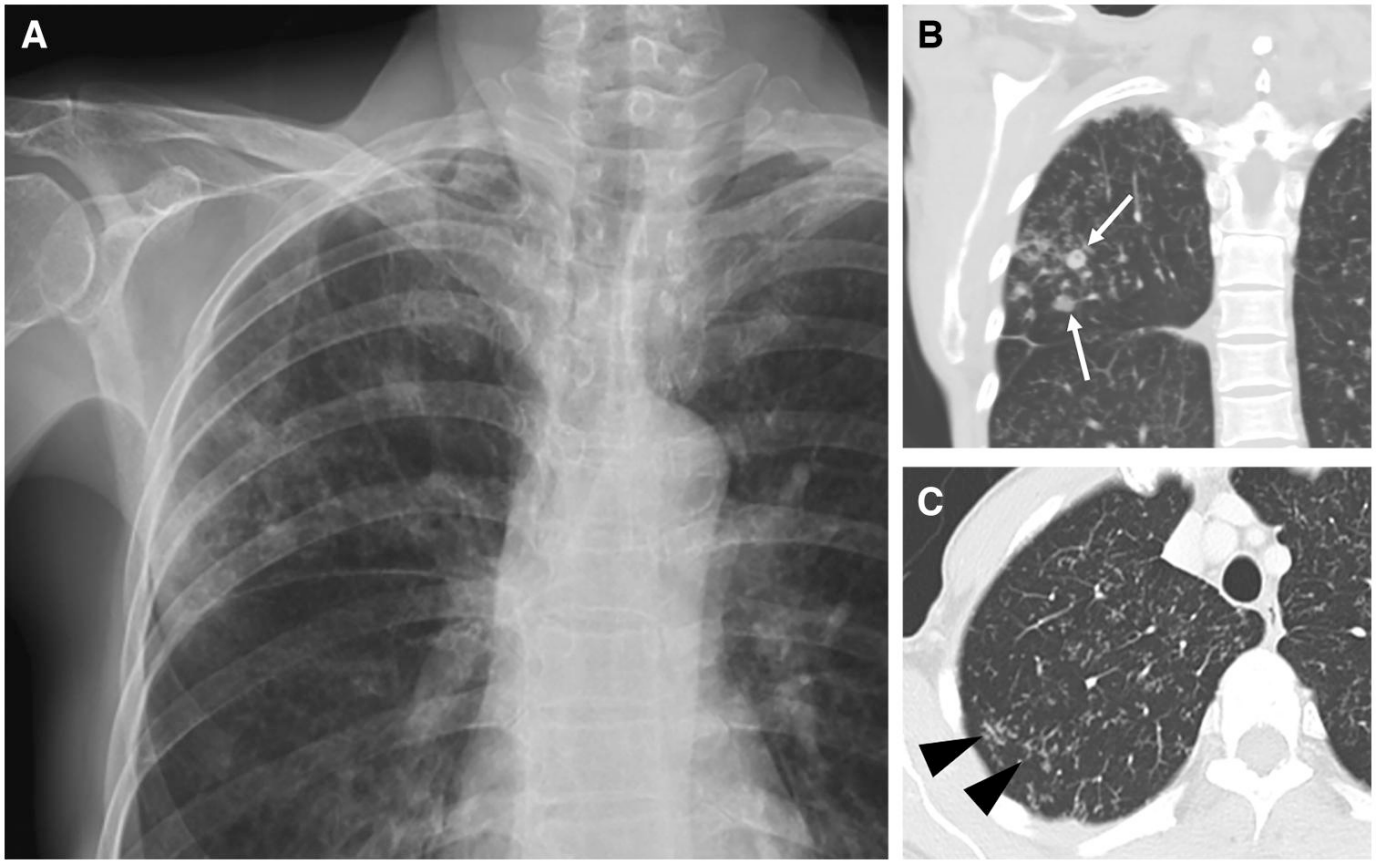
Pulmonary tuberculous nodules in a 61-year-old woman. (A) Frontal chest radiograph and (B) coronal CT image show reticulonodular infiltration in the right upper lobe, with several nodules (white arrows). (C) Axial CT image shows tree-in-bud opacities in central zone as well as in the peripheral posterior aspect of the lung (black arrowheads).

**Figure 4.**
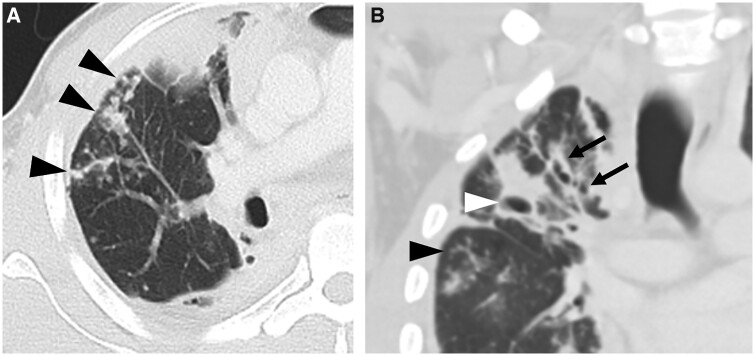
Postprimary TB with tree-in-bud opacities in a 30-year-old man. (A) Axial CT image shows tree-in-bud appearance (black arrowheads) seen as multiple small, centrilobular nodules connected to linear branching opacities. (B) Coronal CT image shows reticulonodular infiltration, cavitary lesion (white arrowhead) and traction bronchiectasis with wall thickening (arrows). Tree-in-bud opacities are also seen (black arrowhead).

In about 5% of patients with active TB, the only chest radiographic finding is a pulmonary nodule called a tuberculoma which if solitary, may mimic tumour. In up to 80% of cases, small satellite nodules may be seen adjacent to the tuberculoma.^27^ While consolidation and cavities may be detected on radiographs, CT is better for characterizing small or subtle lesions, and for identification of satellite nodules (Figure 3). Bronchogenic spread produces a segmental or lobar distribution of well-defined linear branching opacities and multiple centrilobular nodules measuring 2-4 mm in size; resulting in the “tree-in-bud” appearance, typically involving the lower lobes.^22^^,^^24^ CT is the best modality to identify the “tree-in-bud” pattern which is difficult to detect radiographically (Figures 3 and 4).

**Figure 5.**
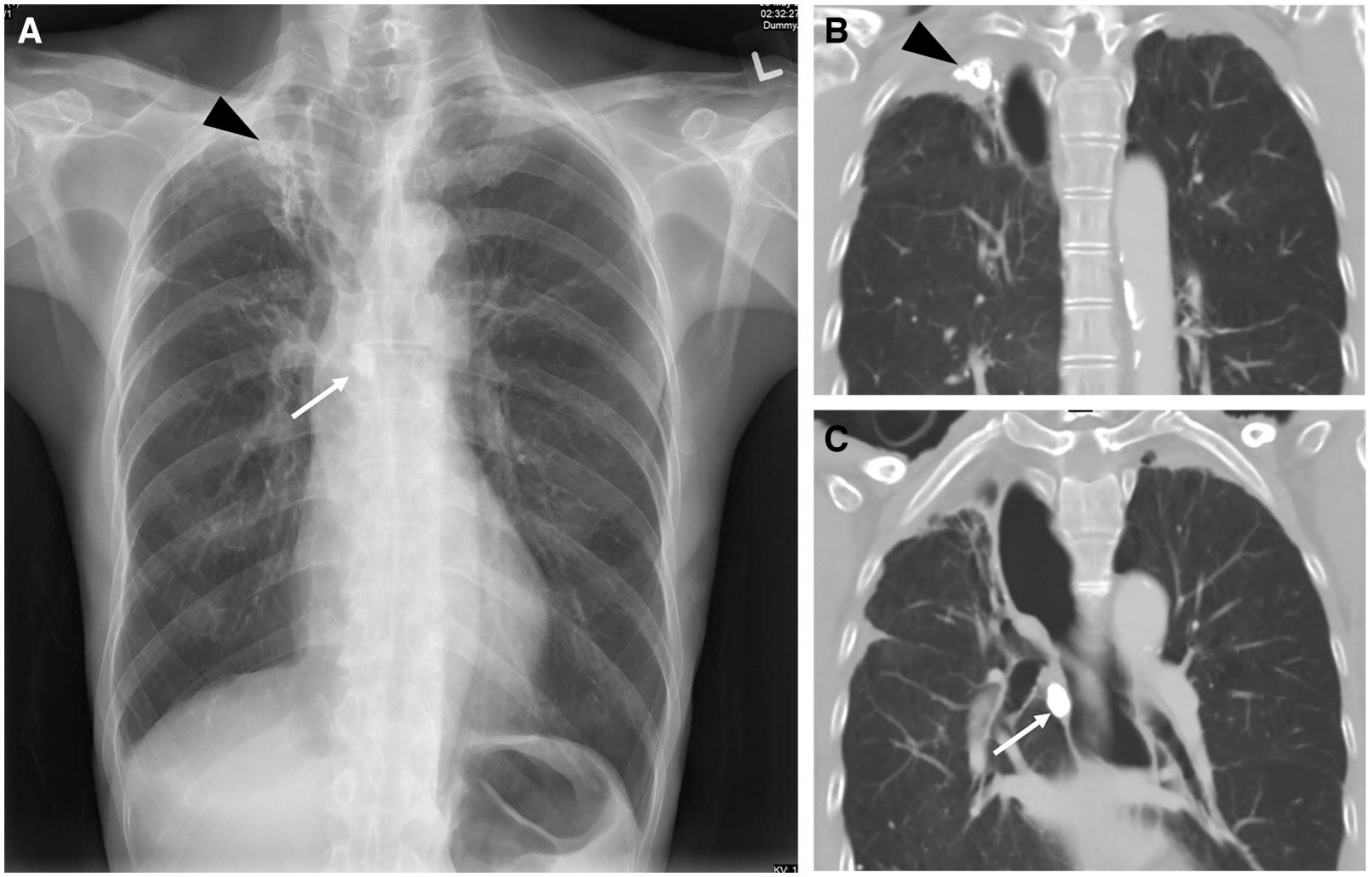
Previous pulmonary TB with Ranke complex in an 84-year-old man. (A) Frontal chest radiograph shows right upper lobe fibrosis (black arrowhead) with right hilar calcification (white arrow). (B and C) Coronal CT images better show the right upper lobe fibrocalcific lesion (black arrowhead) as well as right tracheobronchial calcified lymph node (white arrow).

**Figure 6.**
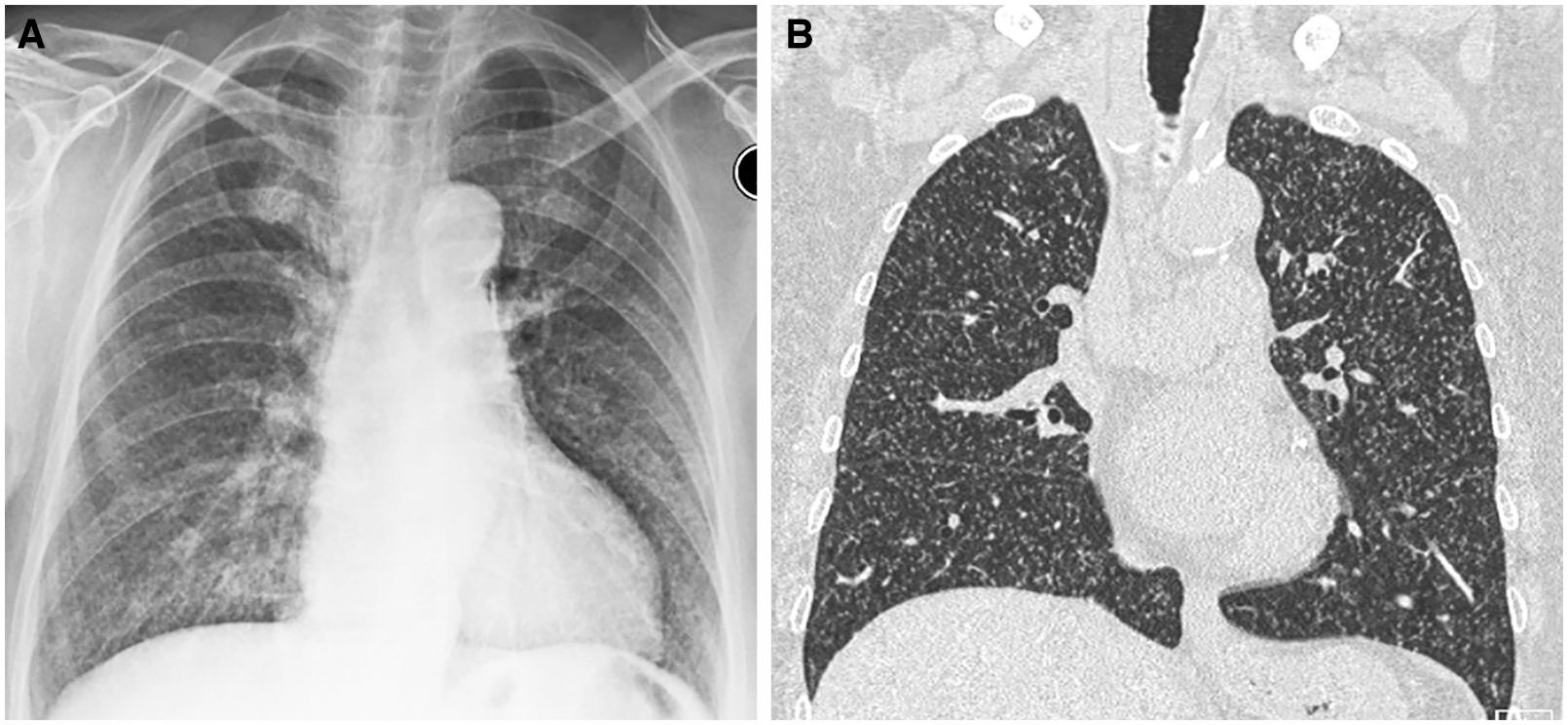
Miliary TB in a 67-year-old woman. (A) Frontal radiograph and (B) coronal CT image show numerous tiny miliary nodules in both lungs.

Lymphatic spread may produce hilar and paratracheal lymphadenopathy, which is typically unilateral and right-sided. Bilateral lymphadenopathy occurs in about one-third of cases.^25^ In classical primary TB, active disease manifests as unilateral hilar lymphadenopathy together with ipsilateral parenchymal consolidation, classically subpleural in location. Sometimes, lymphadenopathy occurs without pulmonary lesions. Radiographically, lymphadenopathy is seen in up to 96% of children and 43% of adults.^25^ Complications of tuberculous lymphadenopathy include obstruction producing consolidation or atelectasis, or fistulation into adjacent bronchus.^28^ Tuberculous lymphadenopathy is best seen on CT as enlarged lymph nodes >2 cm in size, usually with a hypodense centre and rim enhancement.^22^^,^^25^^,^^27^ In two-thirds of cases, lesions regress with massive calcified scar formation.^22^ Finding a Ranke complex is indicative of previous TB (Figure 5). Haematogenous spread results in miliary TB, found not only in lung parenchyma but often concomitantly involves extrapulmonary organs. Miliary TB manifests as numerous tiny nodules of even size, typically 1-3 mm, distributed randomly in the lung parenchyma bilaterally.^22^^,^^25^ Although miliary TB may be detected on radiographs, they are much better seen at an earlier stage on CT (Figure 6).

Whether a patient is drug resistant may affect the patterns and imaging appearances of active TB. Consolidation without cavitation, lymphadenopathy, and pleural effusion represent the most common pattern in patients with primary drug resistance. In patients with multidrug-resistant TB secondary to therapy non-compliance, cavitary lesions are common.^29^ There are certain imaging features that correlate with sputum smear-positive pulmonary TB, namely: cavitation, ground glass opacities, consolidation, nodules, bronchial lesions, lesion multiplicity, and involvement of multiple lung segments and lobes.^30^^,^^31^

**Figure 7.**
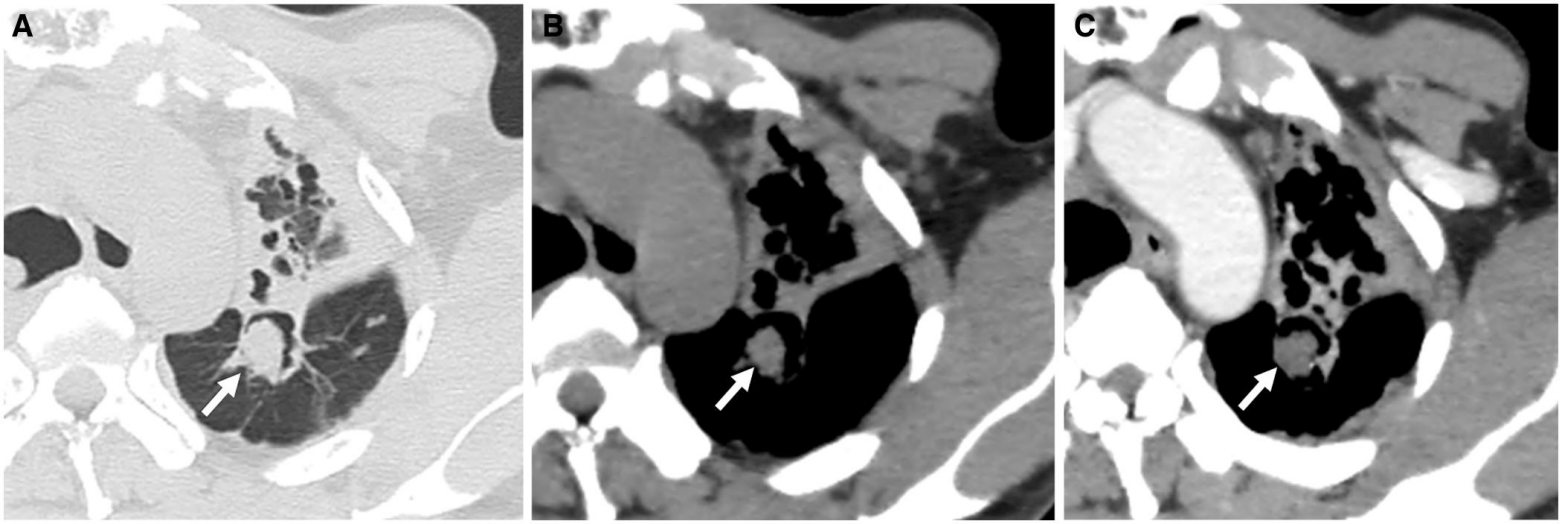
Aspergilloma within a tuberculous cavity in a 69-year-old man. (A–C) Axial CT images show a fungal ball (white arrows) within the upper lobe parenchymal cavity. (A) The lung window image shows a fungal ball in dependent position within a thick-walled cavity. There is an air crescent sign. Note extensive scarring around the cavity. (B) Unenhanced and (C) contrast-enhanced images show that the fungal ball does not enhance. This lack of enhancement helps distinguish a fungal ball from a tumour.

Patients with complications of pulmonary TB such as aspergilloma, Rasmussen aneurysm, bronchiectasis, and systemic hypervascularization may present with life-threatening haemoptysis, often occurring in established rather than active TB.^32^ Pulmonary aspergilloma refers to an *Aspergillus* fungal ball developing in a tuberculous lung cavity. On chest radiographs, it produces the classic “air crescent” sign, consisting of a mobile and gravity-dependent mass within the parenchymal cavity. These features are more optimally seen on CT (Figures 2C and D and 7). In patients unsuitable for surgery, aspergilloma may be treated by intracavitary instillation of antifungal medication which may be performed by CT-guided catheter placement. This treatment mode has a success rate of stopping haemoptysis in 85%-100% of patients and fungal ball elimination in 72.5%.^33^^,^^34^

**Figure 8.**
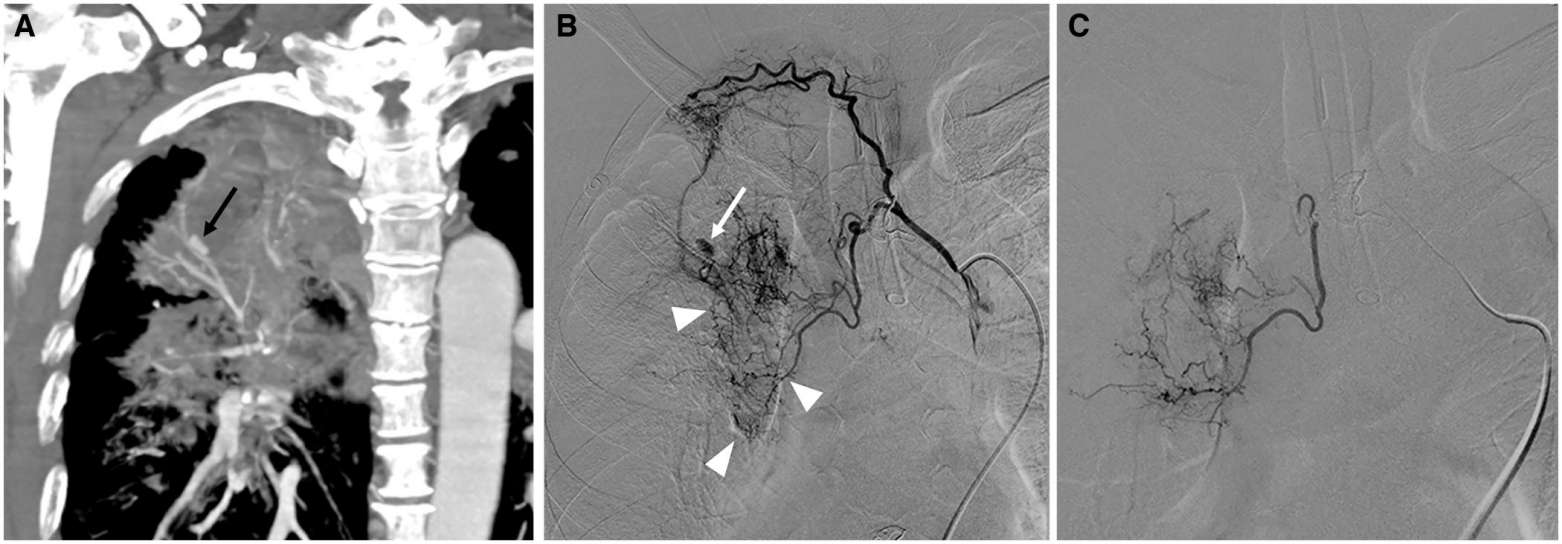
Rasmussen aneurysm in a 65-year-old man who presented with massive haemoptysis. (A) Coronal MIP CT image shows an aneurysm (black arrow) of a branch of the pulmonary artery in the right upper lobe. There is surrounding lung consolidation with areas of contrast extravasation. (B and C) Right intercostobronchial angiography was performed. (B) Preembolization image confirms the aneurysm (white arrow) and increased vascularity of the right upper lobe (white arrowheads). (C) Postembolization image shows occlusion of the aneurysm and reduced surrounding vascularity. (Courtesy of Dr. Tanop Srisuwan, Chiang Mai University, Thailand.)

Rasmussen aneurysm results from weakening of the pulmonary artery wall adjacent to a tuberculous lung parenchymal cavity. Aneurysm rupture may lead to fatal haemoptysis. Rasmussen aneurysm can be diagnosed by contrast-enhanced CT obtained in the arterial phase, which shows signs of active bleeding such as contrast agent extravasation or hyperdense contents within the parenchymal cavity, as well as course and anatomy of responsible vessels. Rasmussen aneurysm can be treated by transcatheter embolization of the pulmonary artery branch together with balloon occlusion^35^ (Figure 8).

## Extrapulmonary TB

In general, extrapulmonary TB is difficult to diagnose both clinically and radiologically, with presenting features being dependent on the organ(s) affected. Hence, TB is well known as the great mimicker of many diseases. These patients often also have specific symptoms related to the affected organ.^5^ However, there is much variability in the frequency and sites of extrapulmonary involvement, depending on various risk factors previously discussed, especially HIV infection/AIDS, specific infective organisms, modes of transmission/spread, and origin from or residence in endemic/developing countries.^36^^,^^37^ After lymph node TB, pleural TB is the second commonest form of extrapulmonary TB.^38^ The other sites of extrapulmonary TB within the thorax are however relatively rare; where TB may affect the heart, chest wall, and breast, frequently mimicking other diseases.

## Pleural TB

### Pathophysiology and clinical features

Pleural TB is found in 3%-5% of patients with TB in developed countries, while frequency is 10 times greater in high-endemic countries. It is particularly prevalent in patients with AIDS.^38^ Pleural effusion may result from the rupture of subpleural pulmonary TB lesions with pleural space communication; or via pulmonary lymphatic spread, followed by acute inflammation and exudation caused by delayed hypersensitivity reaction to tuberculous protein.^22^^,^^39^ Pleural effusion usually develops 3-7 months after initial exposure and is seen in about one-quarter of patients with primary pulmonary TB.^22^^,^^25^ Pleural effusion is less frequently seen in children and in postprimary TB.^22^^,^^25^ As tuberculous pleural effusions usually result from tuberculous protein hypersensitivity rather than frank pleural infection, isolation of *M tuberculosis* from pleural fluid is uncommon. If the results of pleural fluid analysis are not conclusive, pleural biopsy helps increase the diagnostic yield.^22^

### Imaging features

**Figure 9.**
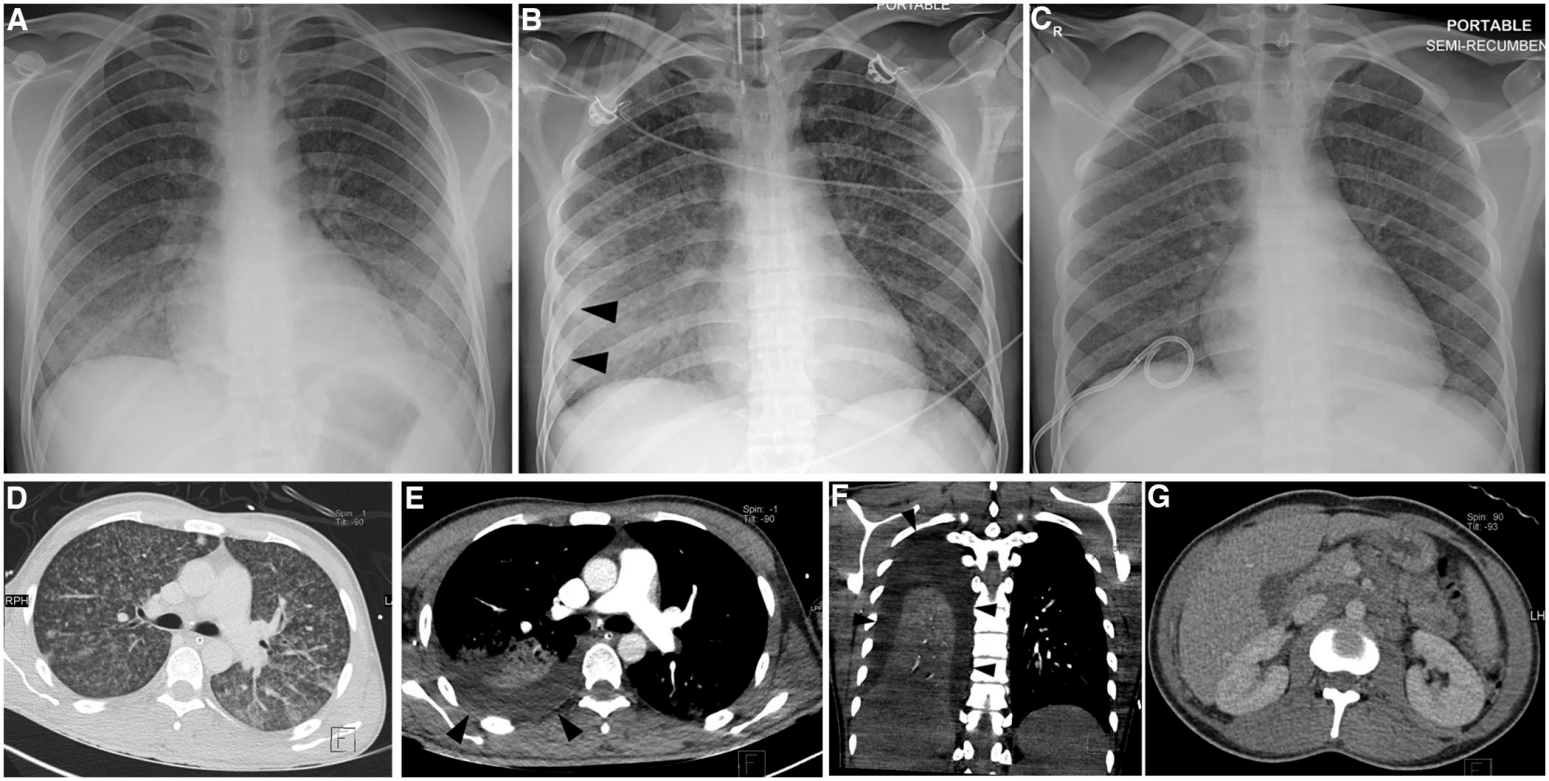
Development of pleural effusion in 24-year-old man with miliary TB. (A) Initial frontal chest radiograph shows multiple tiny nodules in both lung fields, typical of miliary TB. (B) Portable chest radiograph taken 7 days later after the patient’s condition deteriorated shows slight blunting of the right costophrenic angle and right peripheral pleural opacity (black arrowheads). (C) Repeat chest radiograph after drainage catheter insertion shows resolution of right pleural effusion. (D) Initial axial CT image taken in the lung window shows miliary nodules in both lung fields. (E) Corresponding axial and (F) coronal CT images taken 5 days later show right pleural effusion (black arrowheads) and underlying lung atelectasis. (G) Axial contrast-enhanced CT image of the abdomen shows extensive paraaortic, peripancreatic and superior mesenteric lymphadenopathy.

**Figure 10.**
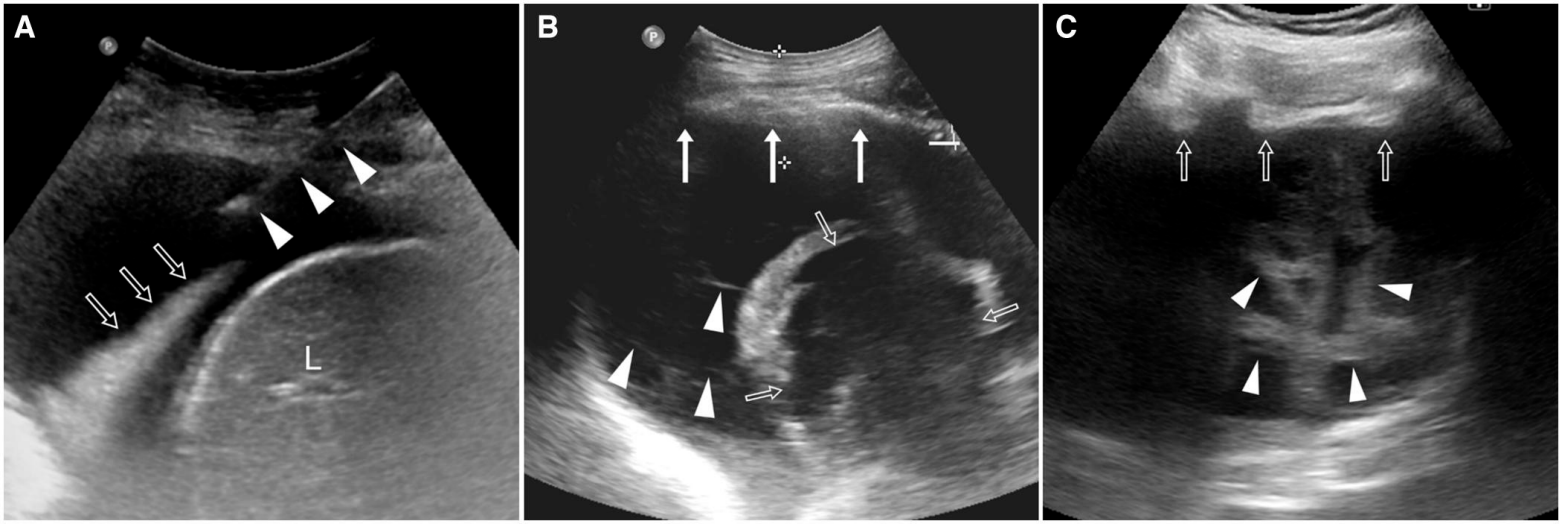
Various appearances of ultrasound (US) imaging in patients with pleural TB. All patients were examined with convex US transducers scanning through the intercostal space. (A) US image in an 85-year-old woman shows homogeneously anechoic effusion with underlying lung atelectasis (open arrows). US-guided thoracentesis was performed (arrowheads indicate needle). L = liver. (B) US image in a 59-year-old man shows smooth pleural thickening (solid arrows) and complex pleural effusion with thin septa (arrowheads). There is also cavitation (open arrows) in the adjacent lung consolidation. (C) US image in a 60-year-old man shows irregular pleural thickening and nodularity (open arrows) with complex thick septations (arrowheads) within the pleural effusion.

**Figure 11.**
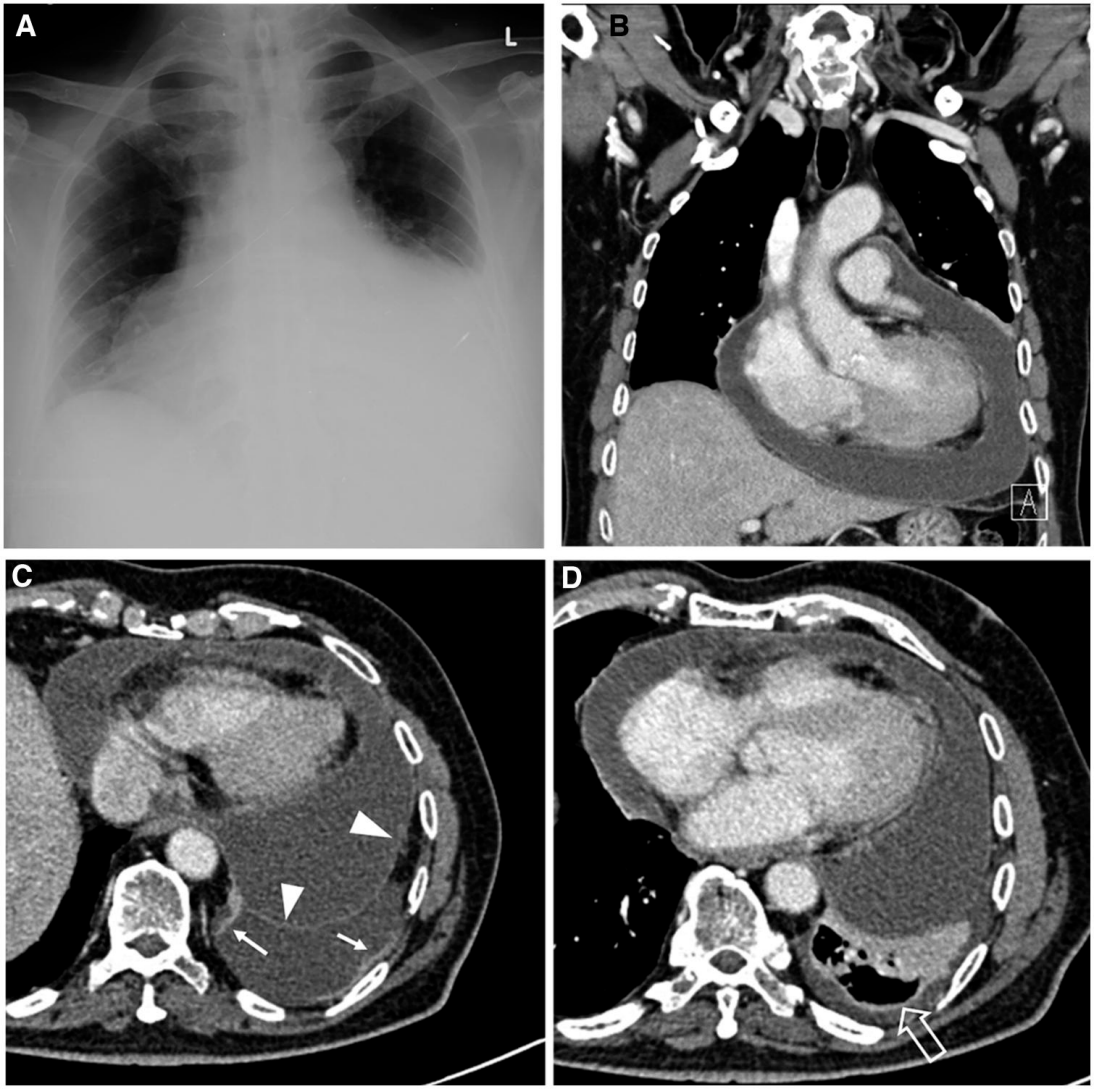
Tuberculous pericarditis and pleuritis in an 81-year-old man. (A) Frontal chest radiograph shows an enlarged globular cardiac shadow with the characteristic “water bottle” appearance. (B) Coronal contrast-enhanced CT image shows pericardial effusion. (C and D) Axial contrast-enhanced CT images show smooth thickening and enhancement of the pericardium (white arrowheads in C), pleural effusion, plaque-like thickening of the parietal pleura (small white arrows in C), thickened visceral pleura (open arrow in D) and compressive atelectasis of the underlying lung.

**Figure 12.**
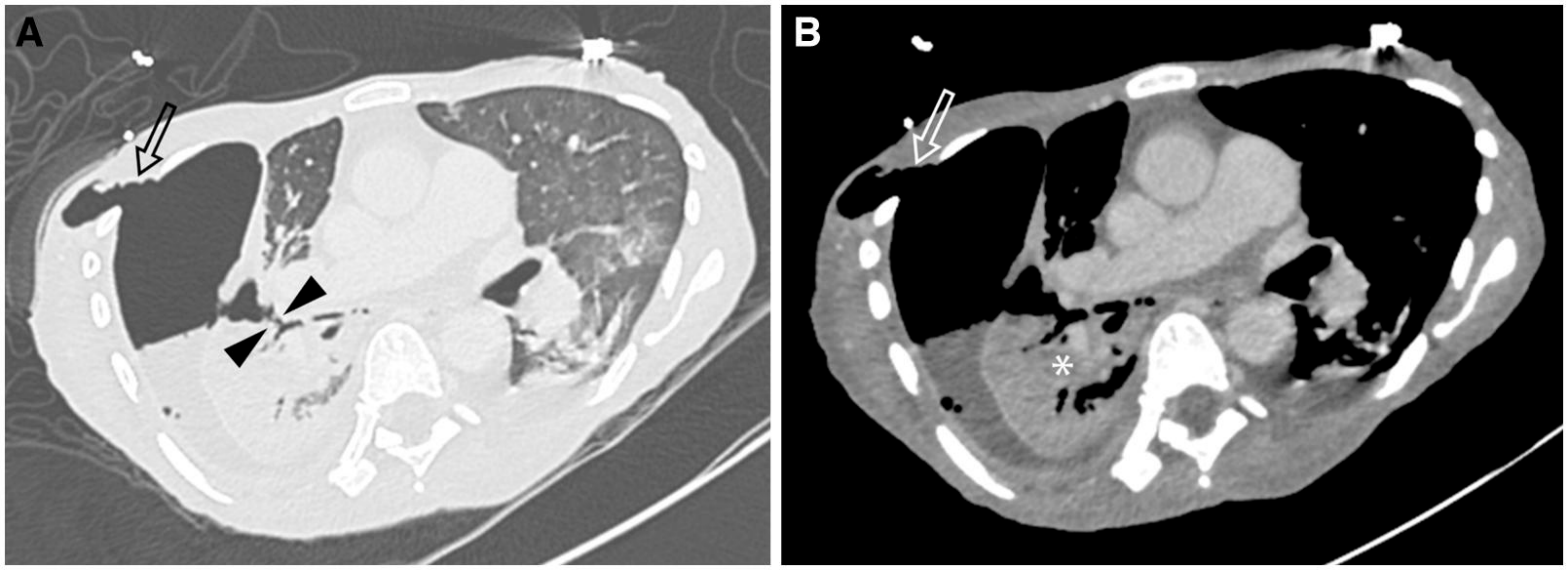
Right empyema necessitans with bronchopleural fistula in a 47-year-old woman with disseminated TB. Axial contrast-enhanced CT images obtained in (A) lung and (B) soft tissue windows show the fistulous tract (arrowheads in A) between right lower lobe bronchus and empyema in right pleural space with extension into the chest wall (arrows in A and B). Soft tissue setting image better shows the right hydropneumothorax with associated smooth pleural thickening and heterogeneous enhancement of the atelectatic right lower lobe (asterisk in B), indicating pneumonia in the atelectatic lung. Small left pleural effusion is also seen.

On imaging, pleural TB is typically unilateral with a variable volume, often of moderate extent. On chest radiographs, concomitant parenchymal involvement is detected in 20%-50% of patients^38^ (Figures 9 and 10). On ultrasonography, pleural effusion may have a variable appearance, ranging from anechoic to hyperechoic, depending on whether empyema has developed.^38^ Besides assessment of features such as effusion volume, septations and pleural thickening, ultrasonography has a role in guiding aspirations of pleural fluid^40^ (Figure 10). Tuberculous empyema is typically loculated, with associated pleural thickening and enhancement.^25^ These latter features are much better appreciated on CT than radiographs (Figure 11). CT is the best imaging technique for comprehensive assessment of the pleura and lung parenchyma (Figures 9 and 11), as well as detecting complications such as bronchopleural fistula.^40^ Empyema necessitans refers to the fistulous extension of pleural empyema into the neighbouring chest wall and surrounding soft tissues, with *M tuberculosis* acknowledged to be the most common cause, accounting for about 70% of cases^41^ (Figure 12). Following treatment and healing, development of residual pleural thickening with calcification may lead to fibrothorax.^22^^,^^25^

## Cardiac TB

### Pathophysiology and clinical features

TB involvement of the heart is generally rare; occurring in up to 2% of immunocompetent patients infected by TB, and rising to 80% of patients with concomitant HIV infection.^42^ Pericardial TB is the most frequent form, with a high mortality rate of approximately 40%.^42^^,^^43^ Pericardial TB may result from retrograde lymphatic spread, haematogenous spread, or rupture from an adjacent pulmonary or pleural lesion.^42^ Modes of clinical presentations include acute pericarditis, myopericarditis and constrictive pericarditis.^43^

### Imaging features

**Figure 13.**
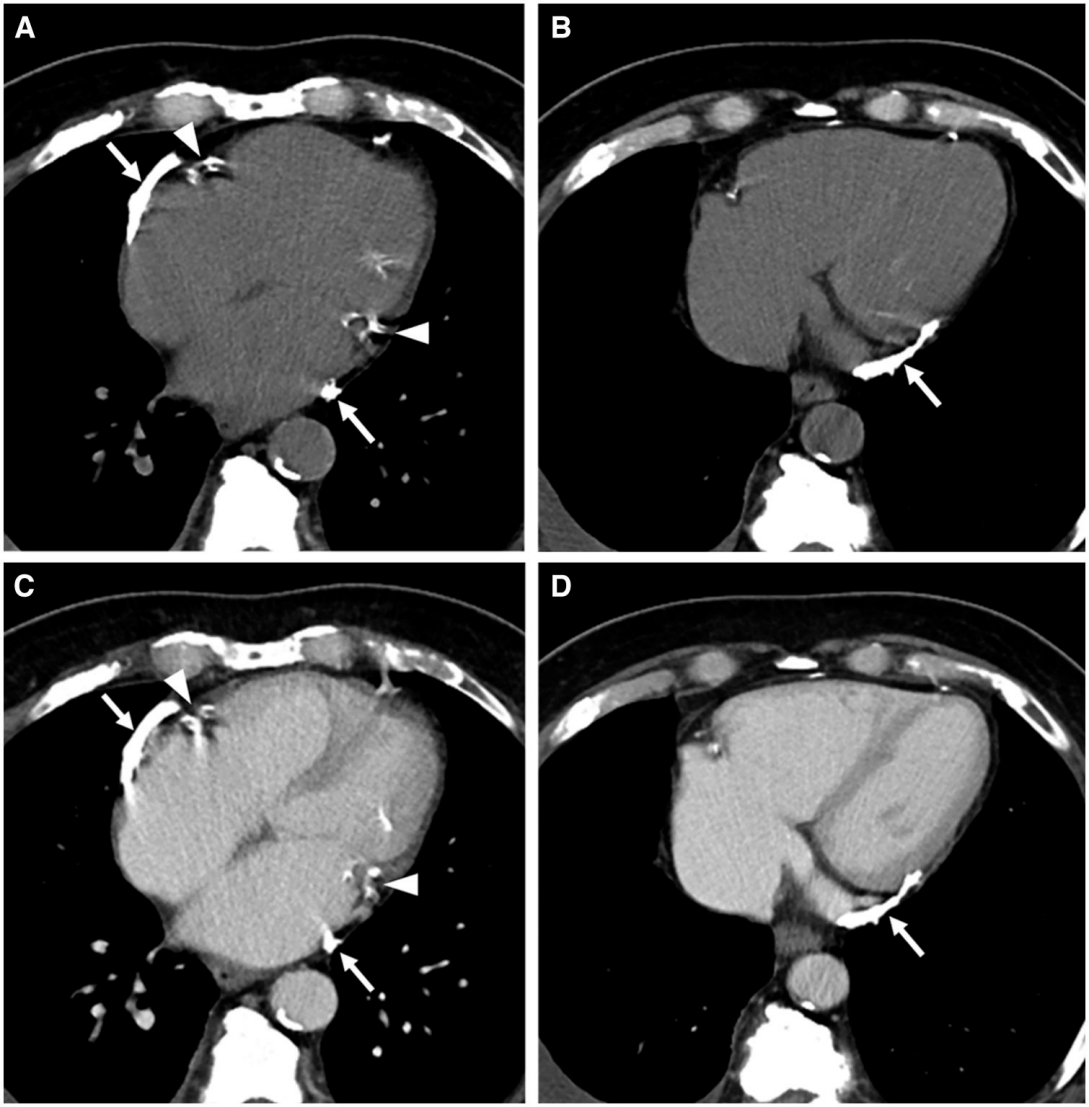
Tuberculous calcific pericarditis in a 79-year-old man. Axial (A and B) unenhanced and (C and D) contrast-enhanced CT images taken at 2 corresponding levels show pericardial calcifications (white arrows) along bilateral atrioventricular grooves with dilatation of right and left atria (bi-atrial enlargement), suggestive of constrictive pericarditis. Note the calcified coronary arteries from atherosclerosis (white arrowheads).

Ultrasonography, CT and MRI are useful in evaluating features of pericarditis such as pericardial effusion, pericardial thickening, and myocardial involvement. On imaging, pericardial TB manifests primarily as pericardial thickening >3 mm, and frequently associated with mediastinal lymphadenopathy. Other features include inferior vena cava distention to >3 cm diameter, pleural effusions that are typically bilateral, pericardial effusions, and development of localized pericardial calcification^25^^,^^37^ (Figure 11). Pericardial calcifications are best shown on CT (Figure 13).

## Chest wall TB

### Pathophysiology and clinical features

Chest wall involvement by TB results either from haematogenous and lymphatic spread, or extension from contiguous mediastinal lymphadenopathy and pleural empyema^44^ (Figure 12). Patients generally present with a fluctuant painless mass without inflammatory signs. Chest wall TB may also present clinically as a palpable breast mass.^44^^,^^45^ The ribs are usually involved, with sternal and clavicular involvement being uncommon.

### Imaging features

**Figure 14.**
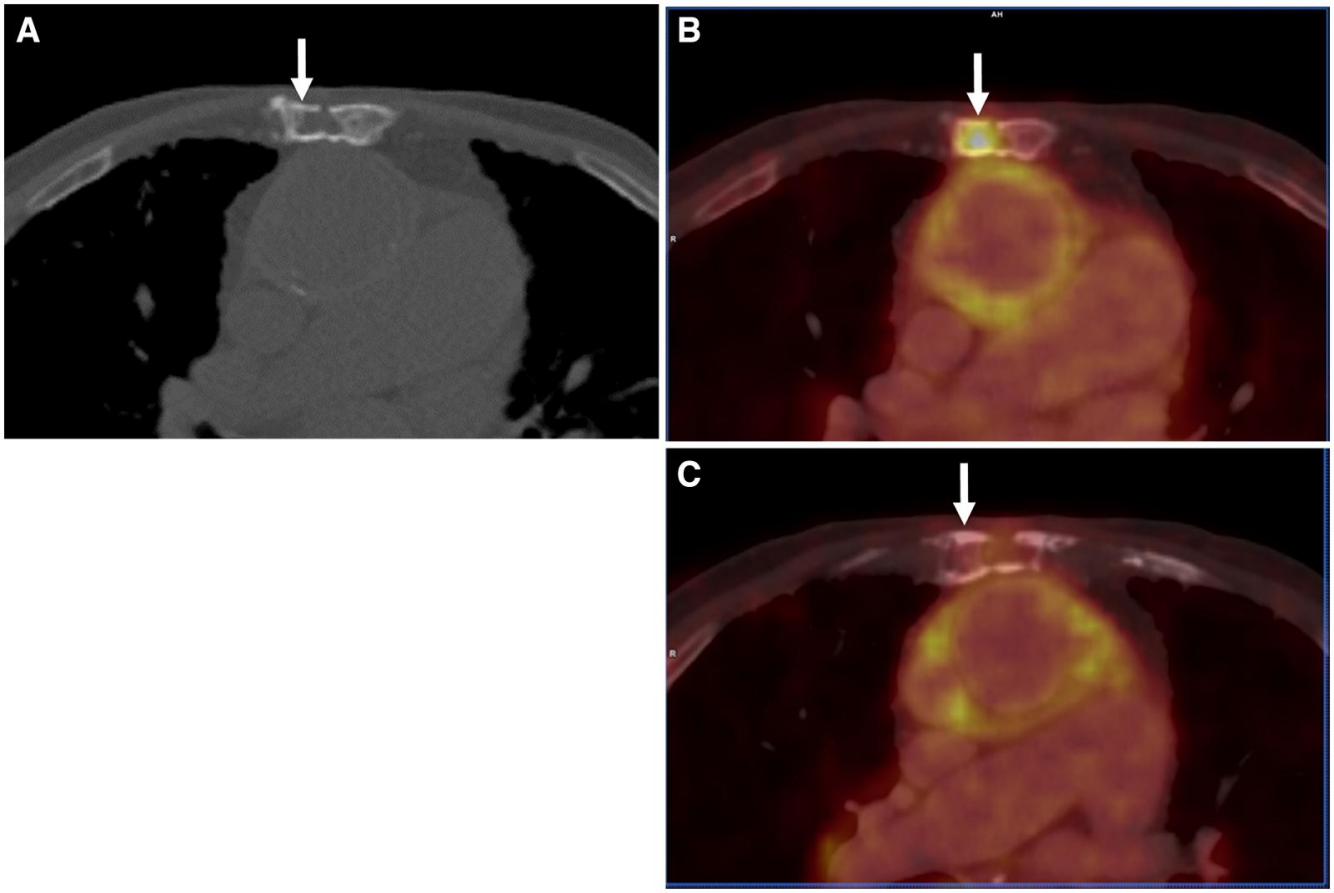
Tuberculous osteomyelitis of the chest wall in an 83-year-old man who had prolonged fever. Axial (A) CT and (B) ^18^F-FDG PET/CT fusion images show a destructive osteolytic lesion in the sternum that has intense metabolic activity (white arrows). (C) Axial PET/CT fusion image taken 8 months later following medical treatment shows regression of uptake in the lesion (white arrow). (Courtesy of Dr Tawika Kaewchur, Chiang Mai University, Thailand.)

On imaging, signs of chest wall TB infection include extensive osteolysis without osteoblastic reaction, and the presence of bone sequestra or fine soft tissue calcifications on radiographs and CT^46^ (Figure 14). An associated cold abscess may be seen on ultrasonography as thick-walled fluid collection containing bone sequestra or heterogeneous mass with necrosis^44^^,^^47^; and appears on CT as fluid collection with rim-enhancing walls.^46^

## Breast TB

### Pathophysiology and clinical features

Breast TB is a very rare manifestation of extrapulmonary TB, with diagnosis being difficult due to non-specific clinical and imaging features. Breast TB may occur by direct inoculation of bacilli through lactiferous ducts, secondary to primary TB infection elsewhere in the body, or rarely, from direct extension from chest wall TB.^48^ The commonest clinical presentation is a painless or painful lump, with inflammatory changes such as skin discolouration, skin ulcer, sinus tract, and nipple discharge being less frequent. Patients with tuberculous lymphadenitis may present clinically with a palpable axillary mass, triggering a search for occult breast cancer.^49^

### Imaging features

**Figure 15.**
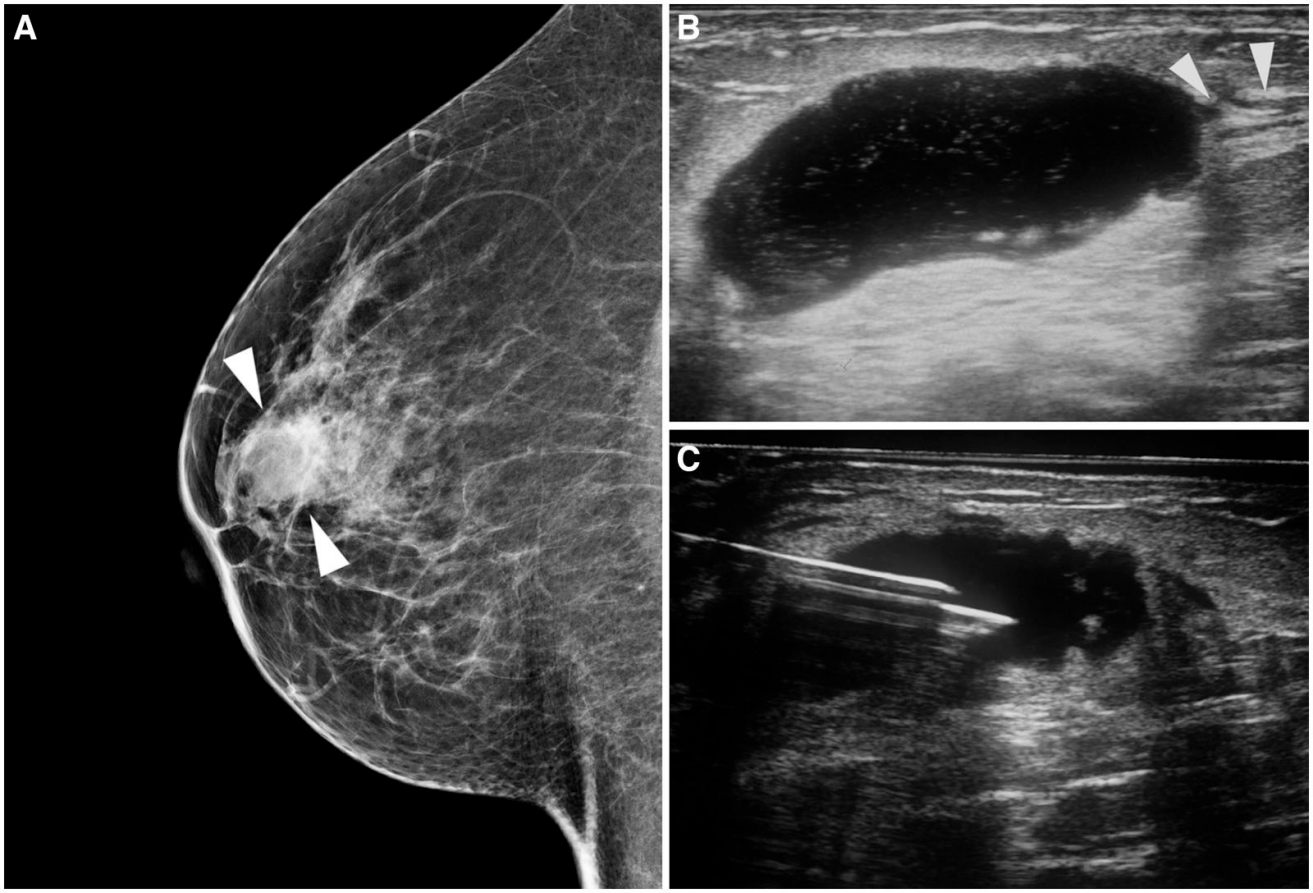
Right tuberculous mastitis in a 36-year-old woman who presented with a palpable breast mass. (A) Right medial-lateral oblique (MLO) mammogram image shows an ill-defined mass (arrowheads) with associated trabecular thickening in the breast. (B) Corresponding ultrasound (US) image shows parenchymal oedema in the right breast with an abscess and fistulous tract (arrowheads). (C) US image shows fine-needle aspiration of the right breast lesion. Histopathology revealed multiple necrotizing epithelioid granulomas with Langhans giant cells amidst dense inflammation. Granulomas showed coalescence with areas of caseation. There was no evidence of intraductal or invasive carcinoma. (Courtesy of Dr Tanvi Jakhi, Mammocare, Mumbai, India.)

**Figure 16.**
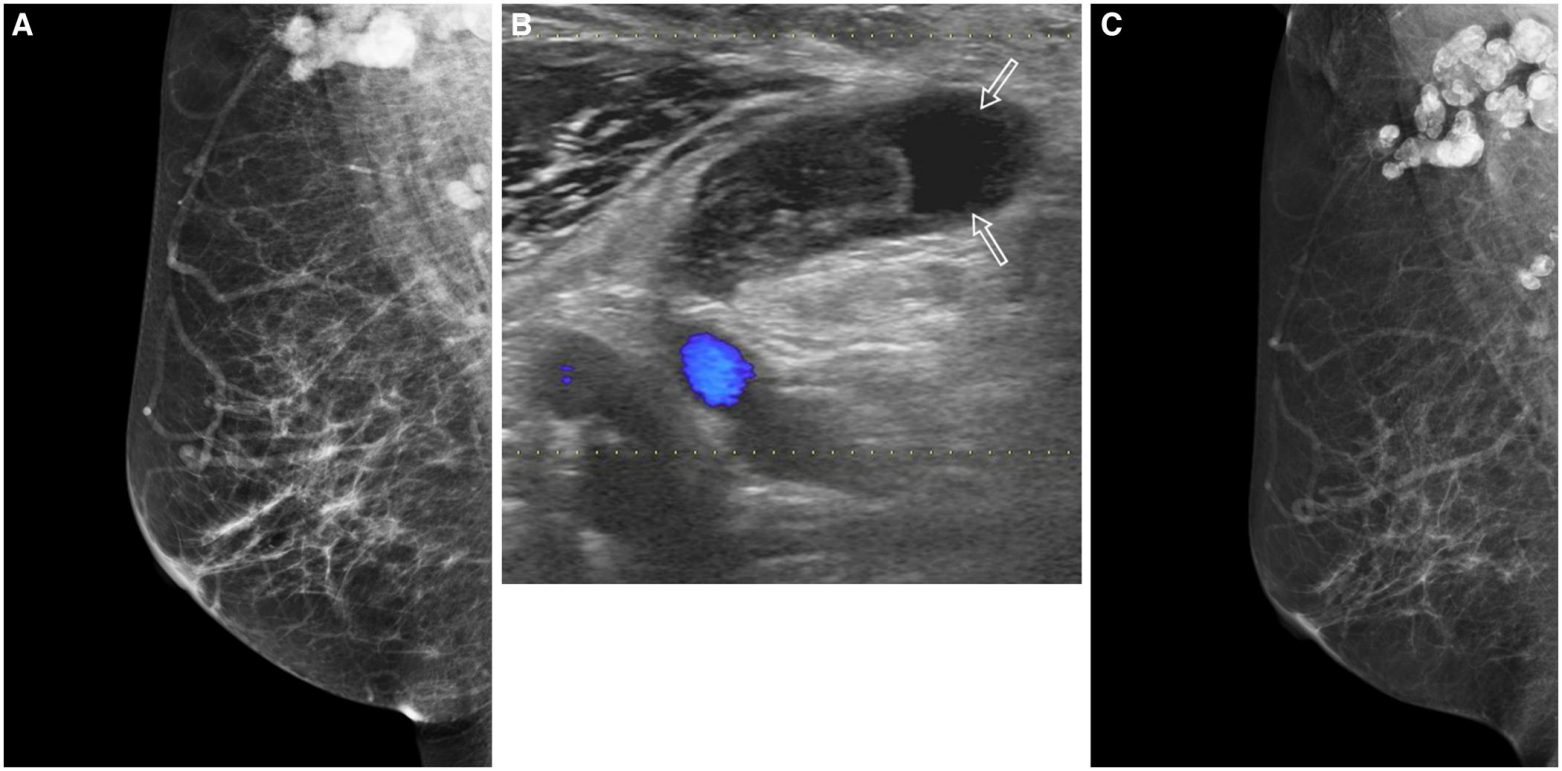
Tuberculous (TB) lymphadenitis in a 56-year-old woman who presented with a palpable right axillary mass. (A) Right medial-lateral oblique (MLO) mammogram image shows multiple enlarged, dense axillary lymph nodes without abnormality in the breast. (B) Corresponding colour Doppler ultrasound (US) image shows an enlarged axillary lymph node with heterogeneous echogenicity and internal cystic component (arrows). US-guided fine-needle aspiration of the internal cystic component showed caseous necrotic material with positive acid-fast bacilli. (C) Right MLO mammogram taken 1 year following anti-TB therapy shows coarse calcifications in the enlarged axillary lymph nodes.

On mammograms, patterns of breast TB consist of the nodular, diffuse, and sclerosing forms, with the nodular form being the most common. The nodule may have well-defined margins initially, with ill-defined spiculated margins later on. The diffuse form is seen as an area of diffusely increased density with skin thickening and oedema. The sclerosing form may appear as an ill-defined irregular dense mass with focal or diffuse skin thickening. All these forms mimic the different appearances of breast carcinoma.^48^^,^^50^ Finding of localized skin thickness and sinus tract associated with an ill-defined breast mass should alert the possibility of breast TB^51^ (Figure 15). On mammograms, tuberculous axillary lymphadenitis is seen as large homogeneously dense lymph nodes with either well- or ill-defined margins, which may be matted. Coarse lymph node calcifications are highly suggestive of previous TB infection^49^ (Figure 16).

## Conclusion

TB remains a major global health issue, particularly in large swathes of the developing world. Although its pathogenesis is fairly well established, there are still many aspects that are not fully understood. The vast majority of patients who are diagnosed with TB infection and who go on to develop TB disease can be successfully treated with standard drug regimens, provided diagnosis is timely enough. Diagnosis and treatment of extrapulmonary TB remain challenging, particularly at less typical sites such as the heart, chest wall, and breast. Radiologists should be familiar with the spectrum of imaging features of TB which can affect both pulmonary and extrapulmonary locations in the thorax.
